# Molecular detection of *Porcine astrovirus* in Sichuan Province, China

**DOI:** 10.1186/s12985-015-0462-6

**Published:** 2016-01-06

**Authors:** Yuhan Cai, Wenqi Yin, Yuanchen Zhou, Bi Li, Lun Ai, Meng Pan, Wanzhu Guo

**Affiliations:** Livestock and Poultry Biological Products Key Laboratory of Sichuan Province, Huashen Veterinary Biological Products Co., LTD, Chengdu, 610299 China; Veterinary Biologicals engineering and technology Research Center of Sichuan Province, Huashen Veterinary Biological Products Co., LTD, Chengdu, 610299 China; Institute of Animal Nutrition, Sichuan Academy of Animal Science, Chengdu, 610299 China; Animal Biotechnology Center, College of Veterinary Medicine, Sichuan Agricultural University, Chengdu, 611134 China

**Keywords:** *Porcine astrovirus*, Molecular detection, Phylogenetic analysis

## Abstract

**Background:**

Porcine astrovirus (PoAstV) is widely distributed worldwide, and is highly prevalent among piglets with or without diarrhea, existing as at least five distinct lineages (PoAstV1–PoAstV5) within the genus *Mamastrovirus*. However, our knowledge of the diversity and epidemiology of PoAstV in China is limited.

**Results:**

In this study, fecal samples from 21/120 (17.5 %) domestic pigs, including 18/100 (18 %) diarrheic and 3/20 (15 %) healthy pigs, and from 1/9 (11.1 %) healthy wild boars tested in Sichuan Province were positive for PoAstV on reverse transcription–PCR. Of the 22 positive samples, 13.6 % were positive for PoAstV only, whereas 40.9 % also contained *Porcine epidemic diarrhea virus* (PEDV), 22.7 % also contained porcine group A rotavirus (PRoVA), and 22.7 % also contained PEDV and PRoVA. A phylogenetic analysis of the RdRp gene revealed genetic heterogeneity among the PoAstV sequences and two lineages were detected in this study, with PoAstV-2 predominant. PoAstV-5 was detected in wild boars for the first time.

**Conclusions:**

PoAstV infections exist in Sichuan Province regardless of the disease status in the pig population, either alone or in combination with other enteric viruses, and may be associated with diarrhea.

**Electronic supplementary material:**

The online version of this article (doi:10.1186/s12985-015-0462-6) contains supplementary material, which is available to authorized users.

## Findings

Astroviruses (AstVs) are members of the family *Astroviridae*. They are single-stranded positive-sense RNA viruses with genomes of approximately 7 kb and spherical, nonenveloped virions of about 30 nm in diameter. The family *Astroviridae* is currently separated into two genera, *Mamastrovirus* (33 species) and *Avastrovirus* (seven species), which infect mammals and birds, respectively. They are generally associated with either mild or severe enteric disease in mammals, but are also found in healthy animals [1, 2]. In addition to their intestinal manifestations, recent studies have reported that AstVs can also cause extraintestinal clinical symptoms in humans, minks, pigs, ducks, and goose embryos [3, 4].

*Porcine astrovirus* (PoAstV) was first reported in 1980 in fecal samples from diarrheal pigs, and PoAstV was isolated in culture in 1990. However, the first molecular characterization of PoAstV was not reported until 2001 [5]. Recently, a number of PoAstV isolates have been detected in several countries and characterized, and at least five distinct PoAstV lineages have been identified since PAstV-5 was first identified in fecal samples from slaughtered pigs in Canada in 2009 [6]. To date, PAstV-5 has been identified in domestic pigs, with or without diarrhea, in five countries, including the USA [3, 7], Sweden [4], Canada [6], China [8], and Croatia [2, 9], whereas no study has reported PAstV-5 in wild boars.

However, our knowledge of the diversity and epidemiology of PoAstV in China is limited, and only three studies have reported the genotypes of PAstV distributed across China. One study identified and characterized a PoAstV strain belonging to PoAstV-2 (HQ647383) from diarrheal domestic pigs in 2009 and another detected PoAstV-1 (GQ914773) in healthy domestic pigs in 2008 [10, 11]. The third study identified and genetically characterized lineages of PAstV-2 (KP747573) and PAstV-5 (KP747574) from healthy domestic piglets in 2015 [8]. In this study, we investigated the infection status and genotypes of PoAstV in domestic pigs and wild boars in Sichuan Province, China.

A total of 120 fecal samples from domestic piglets were collected from 76 piggeries across 10 districts of Sichuan Province during the winter of 2014. Of these samples, 100 were taken from diarrheic piglets on 61 farms with epidemic outbreaks of diarrhea and the remaining 20 were taken from asymptomatic piglets from 15 farms with no history of diarrhea. Nine fecal samples from clinically healthy wild boars were collected from wildlife areas located in the northwest of Sichuan Province in December 2014. All the samples were placed into specimen containers and immediately transported to the laboratory at 4 °C for PCR screening.

The fecal samples were initially examined for the presence of PoAstV with a nested pan-PCR [12] designed to target a conserved region of the RNA-dependent RNA polymerase (RdRp) gene, which has been used previously for the sensitive and specific identification of AstV in mammals, including humans [13], pigs [1, 2, 6, 9, 14], bats [15], birds [16], rodents, shrews, pikas, and weasels [17]. Clinical symptoms of diarrhea are frequently reported to be associated with PEDV, PRoVA, and *Transmissible gastroenteritis virus* (TGEV) infections in piglets in Sichuan Province, and several papers have described the prevalence of coinfections of PEDV, PRoVA, or TGEV with other enteric pathogens [18, 19]. Therefore, the PoAstV-positive samples were also tested for PEDV, TGEV, and PRoVA with reverse transcription (RT)–PCR, as previously described [18].

The PCR products from PoAstV were cloned into the pMDT-19 simple vector (Takara, Dalian, China) for sequencing (Invitrogen, Carlsbad, CA). All PoAstV sequences were first compared with the BLAST program in NCBI (http://blast.ncbi.nlm.nih.gov/Blast.cgi), and then aligned with other known AstV strains with ClustalW (1.6), using the MEGA 6.06 software. The same software was used to construct a phylogenetic tree from the evolutionary distances between the sequences, using the neighbor-joining (NJ) method and the p-distances of the nucleotide sequences. The clustering stability of the NJ tree was evaluated with 1000 bootstrap replicates. The sequences of all the PoAstV-positive samples were submitted to GenBank under accession numbers KT440857–KT440878.

Of the 120 domestic pig fecal samples tested, 21 (17.5 %) were positive for PoAstV, a considerably lower overall PoAstV-positive rate than those determined in the Czech Republic (34.2 %) [14], Canada (79.2 %) [1], and Croatia (89 %) [2] when the same PCR method was used, but similar to the results when other PCR methods were used, identifying PoAstV prevalence rates of 19.4 % in South Korea [20] and 20.8 % in Germany [21]. The positive rates in non-diarrheic and diarrheic pig fecal samples were 15 % (3/20) and 18 % (18/100), respectively. Only one wild boar tested positive for PoAstV (11.1 %, 1/9). These results suggest that PoAstV is present in Sichuan Province, regardless of the disease status of domestic pigs. The low prevalence of PoAstV in wild boars might be partly attributable to the fact that wild pigs are generally more resistant to many diseases than are domesticated pigs and partly to the limited number of samples tested.

Of the 22 PoAstV-positive samples, three (13.6 %) were positive for PoAstV alone, and nine (40.9 %) were positive for PoAstV and PEDV, five (22.7 %) for PoAstV and PRoVA, and five (22.7 %) for PoAstV, PEDV, and PRoVA (Additional file 1: Table S1). No co-infection with PoAstV and TGEV was detected in this study. Based on these co-infection rates, we infer that PoAstV-infected piglets are probably always co-infected with PEDV and/or PRoVA, consistent with previous studies [18, 20–23]. As well as associations between PoAstV and PRoVA or PEDV, co-infections of PoAstV and other diarrheal pathogens, such as TGEV, porcine bocavirus, porcine torovirus, porcine enterovirus 9, porcine sapovirus, or porcine norovirus, have been observed in previous studies [18, 21–24]. However, the exact role of the AstVs, whether alone or in combination with other enteric viruses, in causing diarrhea in pigs is not yet clear.

**Fig. 1:**
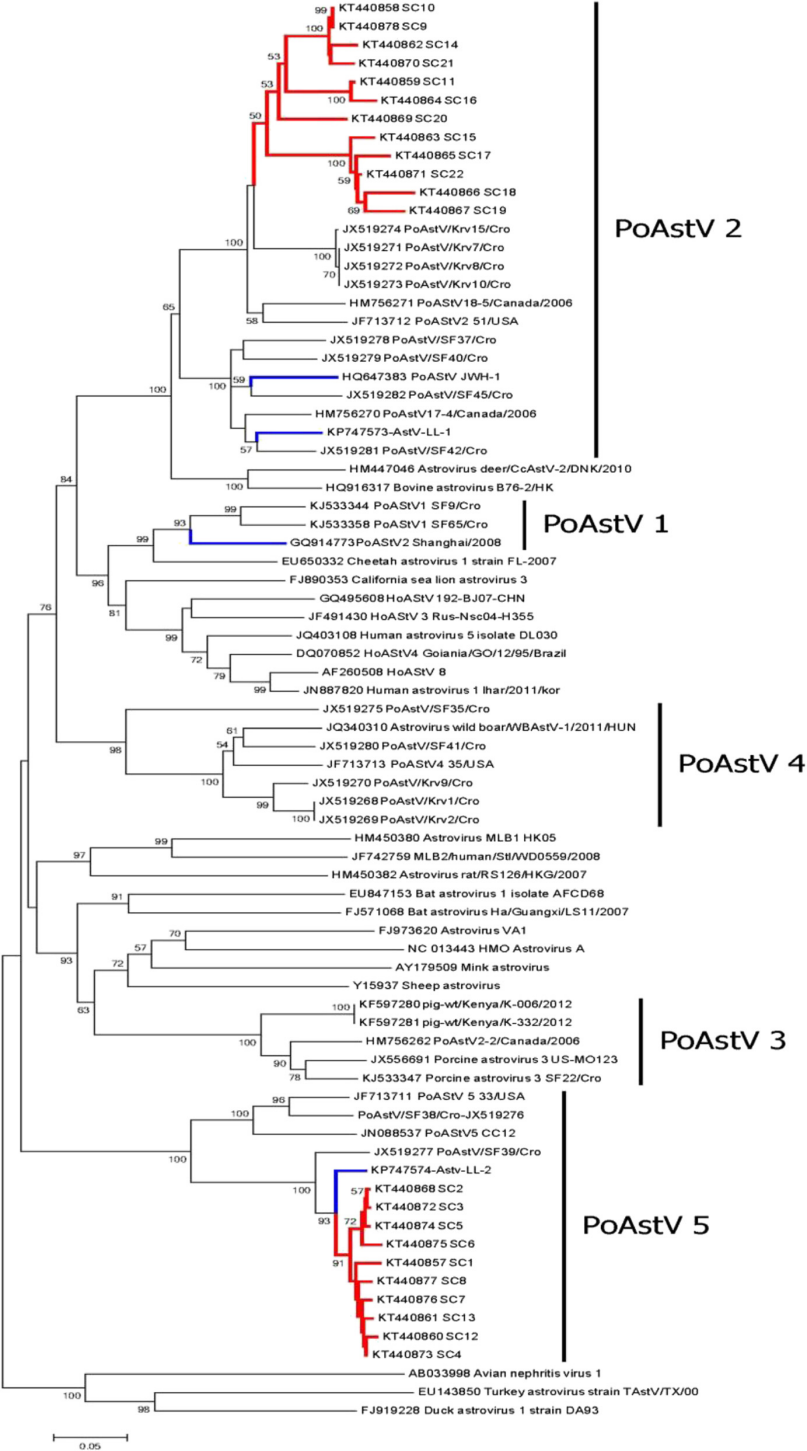
Phylogenetic relationships between the RdRp genes (422 bp) of the porcine astrovirus sequences identified in this study (shown in bold and red) and in previous studies in China (bold and blue) and selected reference sequences (GenBank accession numbers shown with the taxa). The tree was constructed with the neighbor-joining method using the p-distance substitution model, with 1000 bootstrap replicates and a cut-off value of 70 %, with the MEGA 6.06 software. The scale bar represents the number of nucleotide substitutions per site

Genetic typing has been very useful in tracing the evolution and spread of viruses. In this study, we conducted a phylogenetic analysis of a region of the RdRp gene in prototypical PoAstVs, human AstVs, and other AstVs. On the phylogenetic tree (Fig. 1), the PoAstVs were divided into five distinct lineages (PoAstVs 1–5), and some of the PoAstVs are phylogenetically related to other animal and human AstV strains. Recent studies have shown evidence of possible recombination events between porcine and human AstVs [25] and interspecies recombination between porcine and deer AstVs [6]. These findings suggest the zoonotic potential of PoAstV strains.

The 22 PoAstV sequences formed two lineages in the pig population of Sichuan Province (Fig. 1, Additional file 2: Table S2). Twelve strains, comprising two isolates from healthy domestic pigs and 10 isolates from domestic pigs with diarrhea, potentially belonged to PoAstV-2 (54.5 %), sharing 82–99.7 % nucleotide identity, and they formed a single lineage on the phylogenetic tree. Ten strains (45.5 %), including one isolate from a healthy domestic pig, eight isolates from diarrheic domestic pigs, and one isolate from a healthy wild boar, clustered with PoAstV-5, sharing quite high nucleotide similarities of 95–99.4 %. On the phylogenetic tree (Fig. 1), PoAstV-5 has two branches. The Chinese PoAstV-5 strains localized on the same branch as strain JX519277 detected in Croatia in 2011, with 90.5–93.5 % nucleotide identity, and JX519277 was about 73 % identical to the strains on the other branch of PoAstV-5 (JF713711, JX519276, and JN088537). These results suggest that the Chinese PoAstV-5 strains are closely related to JX519277, providing new data for the analysis of PoAstV-5 evolution. However, the origin of the PoAstV-5 strains circulating in China requires further investigation.

Previous studies have reported four PoAstV strains in China: one PoAstV-1 sequence (GQ914773), two PoAstV-2 sequences (HQ647383 and KP747573), and one PoAstV-5 sequence (KP747574). Like previous studies, we also identified PAstV-2 and PAstV-5 in the pig population in Sichuan Province (Fig. 1). The PoAstV-2 sequences detected in our study displayed similarities of 74 and 73.6 % with diversities of 3.4 and 3.5 % to HQ647383 and KP747573, respectively. However, the PoAstV-5 sequences identified in our study shared nucleotide homologies of 94.3–96.7 % with KP747574. These results indicate that three genotypes, PAstV-1, PAstV-2, and PAstV-5, occur in China, with possible antigenic variations among the PoAstVs, as have been reported in several other studies [1, 24, 25]. This is the first time that PoAstV-5 has been detected in wild boars anywhere in the world.

To date, PAstV1–PAstV5 have all been detected in fecal samples from diarrheal or apparently healthy pigs [1, 3, 6, 8]. PAstV5 can also cause congenital tremors [4], and PAstV2 and PAstV4 are found in the blood of pigs [9]. Therefore, more work is required to determine why AstVs are found in both healthy and diarrheic pigs and the clinical manifestations of PAstV infections must be clarified.

In conclusion, this study has demonstrated the presence of PoAstV in domestic pigs and wild boars in Sichuan Province, China, and co-infections of PoAstV with PEDV and/or PRoVA. A phylogenetic analysis of the RdRp gene sequence revealed that PoAstV-2 and PoAstV-5 were isolated in this study. To our knowledge, PoAstV-5 was detected in wild boars for the first time anywhere in the world. PoAstVs play an important role in the evolution and ecology of the *Astroviridae*, and recombination analyses are required to clarify the evolution of PoAstV in Sichuan Province.

## Additional files

Additional file 1: Table S1. Occurrence of PoAstV with other enteric viruses. (DOC 30 kb) Additional file 2: Table S2. Characterization of PoAstV identified in the present study. (DOC 37 kb)
