# Supplementary material for: Molecular detection of Porcine astrovirus in Sichuan Province, China
Source: Virol J. 2016 Jan 6;13:6. doi: 10.1186/s12985-015-0462-6 (PMC4704384; doi:10.1186/s12985-015-0462-6)
Supplement: Additional file 1: Table S1. — Occurrence of PoAstV with other enteric viruses. (DOC 30 kb) [file 12985_2015_462_MOESM1_ESM.doc]

Table S1. Occurrence of PoAstV with other enteric viruses

| Animal species | No. of diarrheic and healthy pigs | RT-PCR detection | | | |
| --- | --- | --- | --- | --- | --- |
|  |  | PoAstV PoAstV+PEDV PoAstV+ PRoVA PoAstV+PEDV+ PRoVA | | | |
| Domestic pigs | Diarrheic (n=100)  Healthy (n=20) | 1(1%)  1(5%) | 9(9%)  0 | 3(3%)  2(10%) | 5(5%)  0 |
| Wild boar | Healthy (n=9) | 1(11.1%) | 0 | 0 | 0 |
| Total | 129 | 3(2.3%) | 9(7.8%) | 5(3.9%) | 5(3.9%) |
