# Supplementary material for: Molecular detection of Porcine astrovirus in Sichuan Province, China
Source: Virol J. 2016 Jan 6;13:6. doi: 10.1186/s12985-015-0462-6 (PMC4704384; doi:10.1186/s12985-015-0462-6)
Supplement: Additional file 2: Table S2. — Characterization of PoAstV identified in the present study. (DOC 37 kb) [file 12985_2015_462_MOESM2_ESM.doc]

Table S2. Characterization of PoAstV identified in the present study

| Name | Accession no. | Origin | State | Genotype | Name | Accession no. | Origin | State | Genotype |
| --- | --- | --- | --- | --- | --- | --- | --- | --- | --- |
| SC1 | KT440857 | Domestic pig | Diarrheic | PoAstV-5 | SC12 | KT440860 | Domestic pig | Diarrheic | PoAstV-5 |
| SC2 | KT440868 | Domestic pig | Diarrheic | PoAstV-5 | SC13 | KT440861 | Domestic pig | Diarrheic | PoAstV-5 |
| SC3 | KT440872 | Domestic pig | Healthy | PoAstV-5 | SC14 | KT440862 | Domestic pig | Healthy | PoAstV-2 |
| SC4 | KT440873 | Domestic pig | Diarrheic | PoAstV-5 | SC15 | KT440863 | Domestic pig | Diarrheic | PoAstV-2 |
| SC5 | KT440874 | Domestic pig | Diarrheic | PoAstV-5 | SC16 | KT440864 | Domestic pig | Diarrheic | PoAstV-2 |
| SC6 | KT440875 | Domestic pig | Diarrheic | PoAstV-5 | SC17 | KT440865 | Domestic pig | Diarrheic | PoAstV-2 |
| SC7 | KT440876 | Domestic pig | Diarrheic | PoAstV-5 | SC18 | KT440866 | Domestic pig | Diarrheic | PoAstV-2 |
| SC8 | KT440877 | Wild boar | Healthy | PoAstV-5 | SC19 | KT440867 | Domestic pig | Healthy | PoAstV-2 |
| SC9 | KT440878 | Domestic pig | Diarrheic | PoAstV-2 | SC20 | KT440869 | Domestic pig | Diarrheic | PoAstV-2 |
| SC10 | KT440858 | Domestic pig | Diarrheic | PoAstV-2 | SC21 | KT440870 | Domestic pig | Diarrheic | PoAstV-2 |
| SC11 | KT440859 | Domestic pig | Diarrheic | PoAstV-2 | SC22 | KT440871 | Domestic pig | Diarrheic | PoAstV-2 |
